# Efficacy and safety of burosumab compared with conventional therapy in patients with X-linked hypophosphatemia: A systematic review

**DOI:** 10.20945/2359-4292-2023-0242

**Published:** 2024-05-17

**Authors:** Manjunath Havalappa Dodamani, Samantha Cheryl Kumar, Samiksha Bhattacharjee, Rohit Barnabas, Sandeep Kumar, Anurag Ranjan Lila, Saba Samad Memon, Manjiri Karlekar, Virendra A. Patil, Tushar R. Bandgar

**Affiliations:** 1 KEM Hospital Seth G.S. Medical College Department of Endocrinology Mumbai Maharashtra India Department of Endocrinology, Seth G.S. Medical College & KEM Hospital, Mumbai, Maharashtra, India; 2 Christian Medical College Department of Child Health Vellore Tamil Nadu India Department of Child Health, Christian Medical College, Vellore, Tamil Nadu, India; 3 Post-graduate Institute of Medical Education and Research Department of Clinical Pharmacology Chandigarh India Department of Clinical Pharmacology, Post-graduate Institute of Medical Education and Research, Chandigarh, India

**Keywords:** Burosumab, calcitriol, phosphorus, X-linked hypophosphatemia

## Abstract

Burosumab, a monoclonal antibody directed against the fibroblast growth factor 23 (FGF23), has been approved for the treatment of X-linked hypophosphatemia (XLH). We conducted a systematic review to compare the efficacy and safety of burosumab versus conventional therapy (phosphorus and calcitriol) on XLH treatment. After a comprehensive literature search on MEDLINE/PubMed and Embase, we found nine studies for inclusion in the analysis. Risk of bias was assessed, and a random-effects model was used to determine the effect size. Clinical, biochemical, and radiological parameters of disease severity before and after treatment were analyzed and expressed in standardized mean difference (SMD). Burosumab resulted in normalization of phosphate homeostasis with an increase in renal tubular phosphate reabsorption and significant resolution of skeletal lesions (change in Thacher's total rickets severity score SMD: −1.46, 95% confidence interval [CI]: −1.76 to −1.17, *p* < 0.001, improvement in deformities, and decline in serum alkaline phosphatase levels [SMD: 130.68, 95% CI: 125.26-136.1, *p* < 0.001)]. Conventional therapy led to similar improvements in all these parameters but to a lower degree. In adults, burosumab normalized phosphorus levels (SMD: 1.23, 95% CI: 0.98-1.47, *p* < 0.001) with resultant clinical improvement. Burosumab treatment was well tolerated, with only mild treatment-related adverse effects. The present review indicates a potential role for burosumab in improving rickets, deformities, and growth in children with XLH. Given its superior efficacy and safety profile, burosumab could be an effective therapeutic option in children. We suggest further studies comparing burosumab versus conventional therapy in children and adults with XLH.

## INTRODUCTION

X-linked hypophosphatemia is the most common genetic cause of rickets and osteomalacia (1). It is caused by loss-of-function mutations in the phosphate-regulating endopeptidase homologue, X-linked (*PHEX*) gene (2). The loss-of-function mutation in the *PHEX* gene results in unregulated secretion of fibroblast growth factor 23 (FGF23) from osteocytes, resulting in permanent renal phosphate wasting and decreased synthesis of the active vitamin D metabolite 1,25 dihydroxycholecalciferol (1,25[OH]_2_D or calcitriol) (3,4). The persistent hypophosphatemia results in defective mineralization, leading to rickets, deformities, stunted growth, abnormal dental development, decreased physical activity in children, and osteomalacia in adults (5-7).

Conventionally, patients with XLH are treated with oral phosphate supplementation in multiple daily doses and active vitamin D (8). If started early, phosphorus and active vitamin D supplementation causes resolution of skeletal lesions and improvement in deformity and growth (9). However, conventional therapy rarely leads to complete resolution of deformities, resulting in need for corrective surgeries and short stature despite optimal treatment (10). Since conventional therapy is administered in multiple daily doses, it may be cumbersome for children and frequently results in poor treatment adherence. Conventional therapy is also associated with a risk of secondary hyperparathyroidism and nephrocalcinosis, requiring meticulous monitoring (11,12). Importantly, oral phosphate supplementation does not address the primary pathophysiology of the disease (*i.e.*, permanent renal phosphorus wasting), and the benefits of conventional therapy are limited by further increases in FGF23 levels (13).

A few randomized trials on the efficacy of burosumab (KRN23; a humanized monoclonal antibody directed against FGF23) have been published in the pediatric and adult population (14-18). These studies revealed the beneficial effects of this novel drug on the resolution of skeletal lesions, normalization of serum phosphorus concentration with decrease in renal phosphorus loss, catch-up growth, and improvement in deformities. However, a complete systematic review of all these studies is lacking. Hence, we conducted a systematic review to address the knowledge gap in the relative efficacy of burosumab versus conventional therapy with phosphate and active vitamin D supplementation in XLH. Estimations of cumulative effect sizes were calculated for both burosumab and conventional therapy to study the magnitude of the treatment effect in XLH.

## MATERIALS AND METHODS

**Figure 1:**
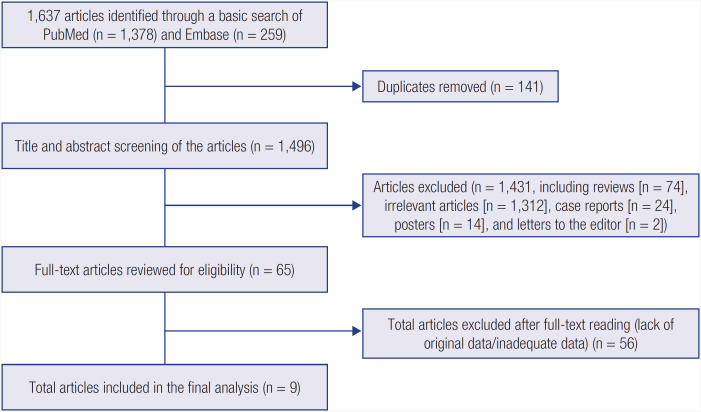
PRISMA flow chart depicting the process of literature search in the systematic review.

The present systematic review was conducted according to the guidelines proposed by the Preferred Reporting Items for Systematic Reviews and Meta-Analyses (PRISMA) statement (Figure 1 and Supplementary Table 1). Once conceptualized, the study protocol was registered with PROSPERO (https://www.crd.york.ac.uk/prospero/) (CRD42022365351).

### Data source

We searched the databases PubMed and Embase for all articles published until January 31^st^, 2023, by using the keywords "Rickets, Hypophosphatemic/therapy"[Mesh]’ OR "Familial Hypophosphatemic Rickets"[Mesh]’ OR "Osteomalacia"[Mesh]’ AND "conventional therapy’ OR "burosumab" OR "KRN23" OR ‘"Phosphorus"[Mesh] OR "Calcitriol/therapeutic use"[Mesh] OR "Antibodies, Monoclonal"[Mesh]" using Cochrane's highly sensitive search strategy for randomized controlled trials (RCTs) (Cochrane Handbook for Systematic Reviews of Interventions, version 6.3, 2022 http://www.cochrane.org/resources/handbook). Two authors (MHD & SCK) searched for studies limited to the English language and manually identified eligible articles derived from the reference lists of relevant articles.

### Eligibility and selection criteria

The initial plan was to include only RCTs. However, due to insufficient numbers of RCTs, we decided to include both open-label prospective observational studies and RCTs describing the safety and efficacy of burosumab and conventional therapy in patients with XLH. Studies on children with rickets aged 1-12 years at the time of enrollment and symptomatic adults with low serum phosphate (18-65 years of age at the time of enrollment) were eligible for inclusion in the systematic review. The studies were screened for the availability of genetic diagnosis (pathogenic variants in the *PHEX* gene (diagnosed by next-generation sequencing/Sangers’ sequencing or, in cases with large deletions, multiplex ligation-dependent probe amplification [MLPA]). The studies were further screened for subjects with family history suggestive of X-linked dominant transmission with elevated FGF23 levels (>30 pg/mL) in case the molecular diagnosis of XLH was not available. We excluded case studies/reports, poster presentations, retrospective studies, and review articles. Two independent reviewers (MHD & SCK) were involved in this process. In case of disagreement between reviewers, we obtained the opinion from the most senior author of the present study (TRB).

### Data extraction and quality assessment

Two reviewers (MHD & SCK) independently extracted the data from studies included in the present review. Disagreements between the authors were resolved by consensus meeting or, if necessary, by a third party (a senior author and the other coauthors). We contacted the articles’ authors (first author and corresponding author) in case of missing data or studies published by similar groups of authors to confirm that each study included a different group of patients. Two authors (MHD & SCK) independently evaluated the methodological quality of the trials. We used the revised Cochrane risk-of-bias tool for randomized trials (RoB 2. BMJ 2019; 366: l4898) to assess the quality of the studies. As per the RoB 2 tool, we graded the risk of bias as "low risk," "some concern," and "high risk" (Supplementary Table 2).

### Definition of outcomes

Outcomes were defined according to the study population. In children, rickets healing is the primary therapeutic objective, so we selected outcomes that offered an objective assessment of rickets improvement with therapy in this group. Hence, the primary outcome was defined as a change in Thacher's total rickets severity (TRS) score from baseline to a post-treatment time point and in the Radiographic Global Impression of Change (RGI-C) score at the end of the study. The TRS score is a validated radiographic measure that assigns a total score ranging from 0 (no rickets) to 10 (severe rickets) based on the sum of the scores obtained by more severely affected wrists (0-4) and knees (0-6) (19). The TRS score is useful to assess the severity of rickets. It can be effectively used to assess the response to treatment. The RGI-C scale is a tool that enables a side-by-side comparison of radiographs obtained before and after treatment and is construed to measure the change in rickets severity. This 7-point ordinal scale ranges from 3 (complete healing) to −3 (severe worsening). We also planned to use the RGI-C scale to assess improvement in deformities (20). Other secondary outcomes were also defined in the pediatric population.

Improvement in osteomalacia with healing of fractures and clinical improvement in bony pain and mobility is a therapeutic goal in adults. Normalization of phosphorus levels in adults is accompanied by improvement in osteomalacia. Hence, the primary outcomes in adults included the increase in serum phosphorus level, the change in the ratio of tubular maximum phosphate reabsorption to the glomerular filtration rate (Tmp/GFR), and the rate of pseudofracture healing. A key secondary endpoint was the change from the first to the last follow-up visit in serum concentrations of alkaline phosphatase, a biochemical marker of rickets severity. Other secondary outcomes were the change in recumbent length/standing height z-scores, normalization of fasting serum phosphorus, and increase in Tmp/GFR from baseline to last visit. In order to assess the safety of burosumab and conventional therapy, relevant and common treatment-related adverse events were tabulated.

### Data analysis

We had initially planned to conduct a meta-analysis using the R Metafor Package, version 4.3.1 (R Foundation for Statistical Computing, Vienna, Austria). However, after extracting the data, we found only one study comparing head-to-head the efficacy of burosumab versus conventional therapy. Thus, for a more comprehensive understanding of the overall effect and to understand the depth of treatment effect in each group, we pulled the data separately.

The primary outcome measure (*i.e.*, the change in TRS score) is presented as a continuous variable (mean and standard deviation [SD]). The secondary endpoints (*i.e.*, change in RGI-C score, TMP/GFR, and serum levels of alkaline phosphate and phosphorus) are presented as continuous values (mean and SD). The summary measure assessed was the standardized mean difference (SMD), presented with 95% confidence intervals (CIs). The SMD is used in studies that report efficacy in terms of measurement of continuous variables, as in the present review. It pools estimates of the effect size of interventions from multiple studies and is calculated as the mean difference divided by the SD. We chose to use the SMD in our study, as it does not depend on the unit of measurement, making it more generalizable. Hence, the SMD was preferred over the mean difference as a measure of effect size.

At first, we computed the SMD for each study using the escalc function based on the necessary information provided by single-arm studies. Next, we created a variance-covariance matrix representing the variances and the covariances of the effect sizes (SMD) for each study. The final step was to calculate a pooled effect size (pooled SMD) by using the rma.mv function in Metafor Package and the variance-covariance matrix created earlier. Since the studies were heterogenous and the single-arm studies contributed to multiple variables, we opted to apply this rma.mv model to calculate the overall effect size for the single-arm studies.

Although the scales changed, we did not measure correlations or assume linearity. We used the chi-square test to assess whether differences observed in the meta-analytic model were compatible with chance alone. Additionally, I^2^ statistic was also planned to detect the percentage of heterogeneity in the meta-analysis.

### Selection of studies

The initial database search yielded 1,637 articles, of which 1431 were excluded after title and abstract screening. A total of 56 articles were further excluded after full-text reading. Finally, nine studies were deemed eligible for inclusion in the systematic review (Figure 1). After data extraction, we found that only one study (Imel and cols.) fulfilled all criteria for inclusion in the meta-analytic model. Since this was the only two-arm study with a head-to-head comparison of burosumab versus conventional therapy, a meta-analysis including other single-arm studies could not be performed. However, we found no systematic reviews of the efficacy and safety of burosumab and decided to conduct the present study as a systematic review. Since most of the relevant studies had a single-arm design, we estimated the pooled effect of burosumab and conventional therapy individually. Here, we provide the forest plots of SMD of primary and secondary outcomes in burosumab and conventional therapy separately to present the overall effect of both interventions in patients with XLH. Although we feel that this type of analysis is not conclusive enough to indicate the best treatment, we expect that the present study will provide guidance to the reader.

## RESULTS

### Characteristics of the patients included in the systematic review

The present review analyzed nine studies, of which five were conducted in pediatric patients with XLH (Table 1). In total, 358 patients with XLH (206 pediatric and 152 adult patients) were interpolated for the analysis. Among the pediatric patients, 109 received burosumab and 97 received conventional therapy (phosphorus plus calcitriol). The combined mean of age at recruitment in studies of pediatric patients was 6.82 ± 2.92 years. Pediatric patients had received conventional therapy for 4.28 ± 3.63 years before enrollment. The combined mean baseline TRS score of patients with rickets was 2.43 ± 1.31.

**Table 1:** Baseline characteristics of the trials included in the present systematic review

Authors (year)	Study subjects	Patients (n)	Age in years (mean ± SD)	Previous treatment (period)	Treatment (dose)	Treatment (duration)	Primary endpoint
Carpenter et al. (2018)	Pediatric patients (rickets)	26	8.7 ± 1.7	Conventional therapy* (7.0 ± 2.1 years)	Burosumab 0.98 mg/kg every 2 weeks	64 weeks	Change in TRS score from baseline to week 64
		26	8.3 ± 2.0		Burosumab 1.5 mg/kg every 4 weeks	64 weeks	
Imel et al. (2019)	Pediatric patients (rickets)	29	5.8 ± 3.4	Conventional therapy (3.3 ± 3.1 years)	Burosumab 0.8-1.2 mg/kg every 2 weeks	64 weeks	Change in rickets severity score (RGI-C) at the end of the study
		32	6.3 ± 3.2		Conventional therapy	64 weeks	
Whyte et al. (2022)	Pediatric patients (rickets)	13	1.7 ± 1.5	Conventional therapy (1.3 ± 1.2 years)	Burosumab 0.8-1.2 mg/kg every 2 weeks	64 weeks	Safety and change in fasting serum phosphorus level
Jin et al. (2022)	Pediatric patients (rickets)	30	6.5 ± 2.6	Most subjects received conventional therapy (2.25 years)	Conventional therapy (calcitriol 20 ng/kg/day)	2 years	Change in TRS score from baseline to the months 12 and 24
		35	5.7 ± 2.9		Conventional therapy (calcitriol 40 ng/kg/day)	2 years	
Namba et al. (2022)	Pediatric patients (rickets)	15	6.7 ± 3.2	Phosphorus (0.3-3 g/day), vitamin D (0.15-4 µg/day)	Burosumab 0.8-1.2 mg/kg every 2 weeks	124 weeks	Safety and changes in TRS and RGI-C scores
Carpenter et al. (2014)	Adults	38	38 ± 13	Not available	Burosumab 0.1-1 mg/kg every 4 weeks	50 days	Safety and changes in serum and urinary mineral biochemistry
Imel et al. (2015)	Adults	28	41.9 ± 13.8	Conventional therapy	Burosumab 0.1-0.6 mg/kg every 4 weeks	22 months	Proportion of subjects achieving fasting serum Pi within the normal range
Insogna et al. (2018)	Adults	68	41.3 ± 11.6	Phosphorus (16.5 ± 10.4 years); active vitamin D (18.2 ± 11 years)	Burosumab 1 mg/kg every 4 weeks (≤90mg)	24 weeks	Change in serum Pi, 1,25(OH)_2_D, and Tmp/GFR from baseline
Cheong et al. (2019)	Adults	6	37.3 (19-57)**	Details regarding prior treatment were unavailable	Burosumab 0.3 mg/kg every 4 weeks	Sequential dose escalation single-dose study (29 days)	Change in serum Pi, 1,25(OH)_2_D, and Tmp/GFR from baseline
		5	31.6 (19-49)**		Burosumab 0.6 mg/kg every 4 weeks		
		7	34.4 (19-57)**		Burosumab 0.9 mg/kg every 4 weeks		

* Conventional therapy includes neutral phosphorus (20-60 mg/kg/day) and calcitriol (20-40 ng/kg/day) or alfacalcidol (40-60 ng/kg/day).

** Mean (range).

Abbreviations: CT, conventional therapy; Pi, inorganic phosphorus; RGI-C, Radiographic Global Impression of Change; Tmp/GFR, ratio of tubular maximum phosphate reabsorption to the glomerular filtration rate; TRS score, Thacher's total rickets severity score.

### Effects of intervention (Tables 2 and 3)

**Table 2:** Effects of intervention: assessment of change in parameters from before to after treatment (change from baseline)

Patients	Parameters	Treatment	SMD (95% CI)
Pediatric	Thacher's total rickets severity score	Burosumab	-1.46 (-1.76; −1.17)
		Phosphorus and calcitriol	-0.86 (-1.45; −0.28)
	RGI-C score	Burosumab	1.83 (1.53; 2.14)
	RGI-C (deformity) score	Burosumab	1.10 (0.71; 1.49)
	Height SDS	Burosumab	0.16 (-0.11; 0.44)
		Phosphorus and calcitriol	0.06 (-0.17; 0.29)
	Phosphorus, mg/dL	Burosumab	1.83 (1.53; 2.14)
	Tmp/GFR	Burosumab	1.05 (0.7; 1.4)
	ALP, U/L	Burosumab	130.68 (125.26; 136.1)
		Phosphorus and calcitriol	26.21 (21.24; 31.18)

Abbreviations: ALP, alkaline phosphatase; CI, confidence interval; RGI-C, Radiographic Global Impression of Change; SMD, standardized mean difference; Tmp/GFR, ratio of tubular maximum phosphate reabsorption to the glomerular filtration rate.

**Table 3:** Effects of burosumab in adults with X-linked hypophosphatemia: assessment of change in parameters from before to after treatment (change from baseline)

Parameters	SMD (95% CI)
Phosphorus, mg/dL	1.23 (0.98; 1.47)
Tmp/GFR	1.09 (0.88; 1.29)

Abbreviations: CI, confidence interval; SMD, standardized mean difference; Tmp/GFR, ratio of tubular maximum phosphate reabsorption to the glomerular filtration rate.

#### Primary outcomes – pediatric group

##### Change in Thacher's TRS score.

**Figure 2:**
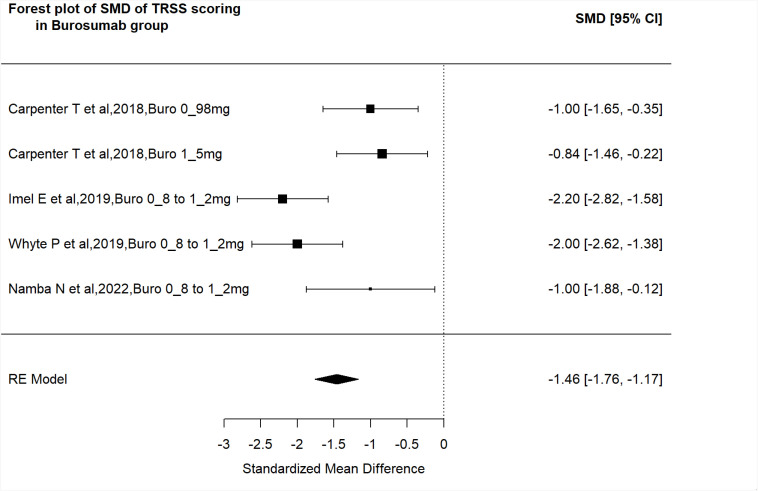
Forest plot of the included studies pooled together using a random-effects model to assess the changes in Thacher's total rickets severity (TRS) score in the burosumab group before and after treatment. The included studies are indicated by the first author and year of publication. The size of each box is proportional to the weight of the corresponding study in the analysis, and the lines represent the 95% confidence interval (CIs). The diamond shape represents the pooled standardized mean difference, and its width represents the corresponding 95% CI.

Five studies (burosumab studies: Carpenter and cols. [2018], Imel and cols. [2019], Whyte and cols. [2019], Namba and cols. [2022]; conventional therapy studies: Jin and cols. [2022] and Imel and cols. [2019]) described the improvement in rickets severity using the change in TRS scores. The effect of individual studies was pooled in a random-effects model using the rma function and the corresponding forest plot was created with the forest plot function, both in the Metafor Package. As mentioned earlier, we preferred SMD over mean difference as a measure of treatment effect, since SMD is more generalizable (21). The pretreatment and post-treatment data from the studies are shown in Figure 2. On the random-effects model, the SMD for burosumab was −1.46 (95% CI: −1.76 to −1.17, p < 0.001), indicating a significant favorable effect of this drug in improving rickets on radiograph (Figure 2). Conventional therapy also had a favorable effect on the resolution of skeletal lesions, with a SMD of −0.86 (95% CI: −1.45 to −0.28, p < 0.001).

##### RGI-C score.

Four studies (Carpenter and cols. [2018], Imel and cols. [2019], Whyte and cols. [2019], and Namba and cols. [2022]) reported the intervention effect as RGI-C scores based on post-treatment radiographs. The RGI-C score SMD was 1.83 (95% CI: 1.53-2.14, p < 0.001) in patients with XLH treated with burosumab. Only one study (Imel and cols. [2019]) reported the RGI-C score in the conventional-therapy group (0.8 ± 0.2).

##### Change in deformity score (RGI-C scores).

Three studies (Carpenter and cols. [2018], Imel and cols. [2019], and Whyte and cols. [2019]) described the effect of burosumab in improving deformity using RGI-C scores. The SMD for the deformity score was 1.1 (95% CI: 0.71-1.49, p < 0.001).

#### Secondary outcomes – pediatric group

##### Change in height standard deviation score (SDS).

Three studies (burosumab group: Carpenter and cols. [2018] and Imel and cols. [2019]; conventional-therapy group: Imel and cols. [2019] and Jin and cols. [2019]) reported improvement in recumbent length/height after treatment relative to baseline. On random-effects model analysis, the SMD was 0.16 (95% CI: −0.11-0.44, p = 0.25) in the burosumab group and 0.06 (95% CI: −0.17-0.29, p = 0.61) in the conventional-therapy group.

##### Change in serum phosphorus level.

Four studies (Carpenter and cols. [2018], Imel and cols. [2019], Whyte and cols. [2019], and Namba and cols. [2022]) reported an increase in serum phosphorus level after burosumab treatment. On random-effects model analysis, the SMD in the burosumab group was 1.83 (95% CI: 1.53-2.14), indicating that burosumab increased serum phosphorus levels.

##### Change in Tmp/GFR.

Two studies (Carpenter and cols. [2018] and Imel and cols. [2019]) described a positive effect of burosumab on Tmp/GFR, a measure of phosphorus reabsorption by renal tubules. The present review revealed a SMD of 1.05 (95% CI: 0.7-1.4) in the burosumab group.

##### Change in serum alkaline phosphatase level.

**Figure 3:**
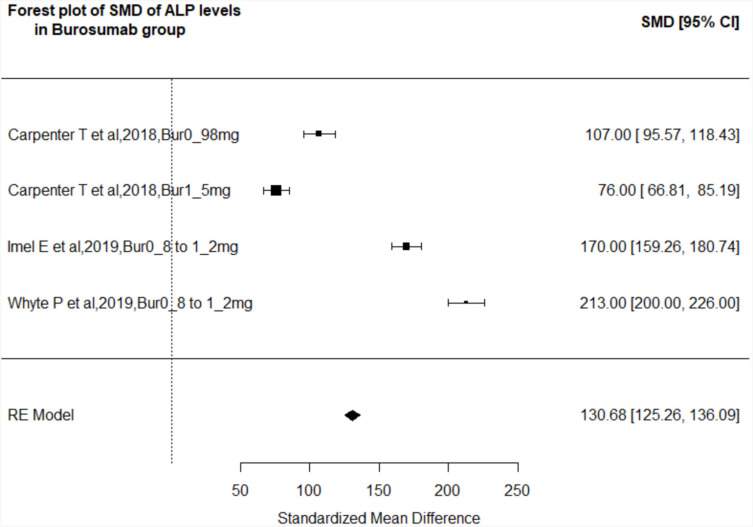
Forest plot of the included studies pooled together using a random-effects model to assess the change in ALP levels in the burosumab group (pediatric population) before and after treatment. The size of each box is proportional to the weight of the corresponding study in the analysis, and the lines represent the 95% confidence intervals (CIs). The diamond shape represents the pooled standardized mean difference, and its width represents the corresponding 95% CI.

Five studies (burosumab: Carpenter and cols. [2018], Imel and cols. [2019], and Whyte and cols. [2019]; conventional therapy: Imel and cols. [2019] and Jin and cols. [2019] (16,22) described pretreatment to post-treatment changes in ALP levels. On random-effects model analysis, the SMD in the burosumab group was 130 (95% CI: 125.26-136.1, p < 0.001), indicating that burosumab had a significant beneficial effect in improving rickets (Figure 3). Of note, conventional therapy also showed favorable effects, with a SMD of 26.21 (95% CI: 21.24-31.18, p < 0.001).

#### Adult population

##### Change in serum phosphorus level.

Adult patients with hypophosphatemic osteomalacia due to XLH were also included in the present systematic review to determine the efficacy of burosumab in this population. Three studies (Carpenter and cols. [2018], Imel and cols. [2019], and Whyte and cols. [2019]) assessed changes in phosphorus levels (normalization) after burosumab treatment was started (15-17). The SMD was 1.23 (95% CI: 0.98-1.47, p < 0.001), indicating a significant beneficial effect of burosumab in normalizing phosphorus levels.

##### Change in Tmp/GFR.

Renal handling of phosphorus is impaired in XLH, leading to isolated loss of phosphorus in urine. Four studies (Carpenter and cols. [2014], Imel and cols. [2015], Insogna and cols. [2018], and Cheong and cols. [2019]) showed effects of burosumab on Tmp/GFR in adults (14,18,23,24). On random-effects model analysis, the SMD was 1.09 (95% CI: 0.88-1.29, p < 0.001) indicating that burosumab significantly improved renal reabsorption of phosphorus.

##### Rate of baseline fracture healing.

Only one study (Insogna and cols. [2019]) reported this outcome in the burosumab group (40% or 26 out of 65 patients).

### Treatment-emergent adverse drug reactions

Injection site reactions with burosumab were reported in 50.85% (interquartile range [IQR]: 40.5%-58.55%) of the pediatric patients and 11.8% (IQR: 17.85%-12.1%) of the adult patients across three studies (Carpenter and cols. [2014], Imel and cols. [2015], and Insogna and cols. [2018]). Secondary hyperparathyroidism was observed in 30% and 20% of the patients in the low-dose and high-dose calcitriol arms, respectively, in patients receiving conventional therapy in the study by Jin and cols. (2022). None of the studies reported secondary hyperparathyroidism in patients with XLH treated with burosumab. Similarly, none of the patients developed hypercalciuria after treatment with burosumab. Studies with conventional therapy did not provide data on the incidence of hypercalciuria after treatment. New-onset dental abscesses were reported in 27.6%, 54.5%, and 53% of the patients receiving burosumab in the studies by Imel E and cols. (2019), Whyte and cols. (2019), and in the age group above 5 years in the study by Ward and cols (2022), respectively. In the age group below 5 years in the study by Ward and cols. (2022), none of the patients developed dental abscess. The proportion of patients with post-treatment dental abscess in the conventional-therapy group was 9.4% and 25% in the studies by Imel and cols. (2019) and Ward and cols. (2022), respectively.

### Heterogeneity

The model output showed the following findings:

- Tau^2 (estimated amount of total heterogeneity): 0.309 (standard error [SE] = 0.302)
- Tau (square root of estimated tau^2 value): 0.556
- I^2 (total heterogeneity/total variability): 72.92%
- H^2 (total variability/sampling variability): 3.69
- Test for heterogeneity: Q (df = 4) = 15.217, p = 0.004.

These results indicate significant heterogeneity (I^2^ = 72.92%), supporting our decision of not submitting the data to meta-analysis.

### Publication bias

**Supplementary Figure 1:**
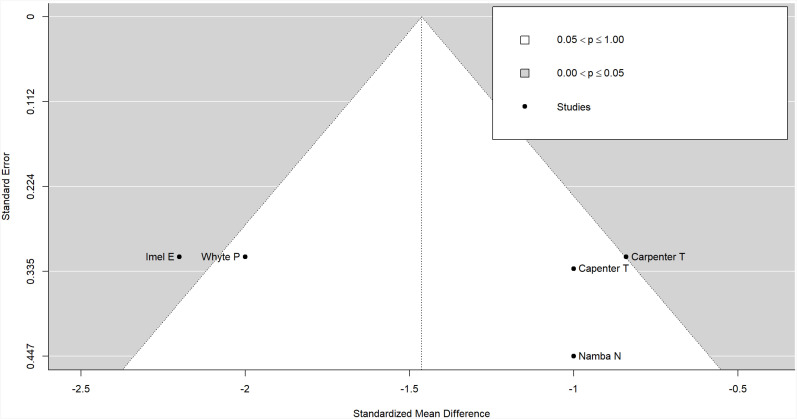
Funnel plot of five studies, indicating that publication bias in these studies could not be excluded.

Based on the pooled estimate and rma.mv model, we generated a funnel plot (Supplementary Figure 1) to identify publication bias. As indicated by the plot, publication bias could not be ruled out.

## DISCUSSION

Burosumab, a novel drug, has become an effective option in the therapeutic armamentarium for patients with XLH. The therapeutic efficacy and safety of burosumab versus conventional therapy were assessed in the present review. The review illustrated that the inhibition of FGF23 activity with burosumab normalized phosphate homeostasis with increase in renal tubular phosphate reabsorption, significant resolution of skeletal lesions, improvement in deformity, and greater decline in ALP levels. Conventional therapy also led to improvement in all these parameters but to a lower degree. In adults, burosumab normalized phosphorus levels with resultant clinical improvement. Burosumab was well tolerated with only mild treatment-related adverse effects.

The studies analyzed in the present review included children who presented with rickets at the age of 1-12 years and adults with osteomalacia. We observed that the children had persistent skeletal changes of rickets at the time of enrollment in spite of the fact that many had received conventional therapy for an adequate duration (16,17). The 2019 study by Imel and cols. showed a mean TRS score of 3.2 ± 1.1 after 3 years of conventional therapy in their study population. Similarly, the 2019 study by Whyte and cols. showed a mean baseline TRS score of 2.9 ± 1.4 in children treated with conventional therapy for 1.3 years. These observations raise questions about the effectiveness of oral phosphorus and active vitamin D in completely resolving the skeletal changes of rickets. In contrast, burosumab induced normalization of phosphorus levels resulting in a significant decrease in TRS score and alkaline phosphatase levels in children who were already receiving conventional therapy. The RGI-C scores were suggestive of substantial healing of rickets with burosumab in the current review. A height benefit was noted but was not significant in any of the two groups. It is possible that the studies’ follow-up duration was not long enough to identify the beneficial effects of burosumab on growth, or the time between the onset of rickets and initiation of burosumab was too long. Nephrocalcinosis and secondary hyperparathyroidism were not observed with burosumab.

In addition to the included studies, we searched for other studies in the literature to support the present review. Our review of the supporting literature indicates that phosphorus supplementation plus vitamin D (ergocalciferol or calcitriol) induces mineralization of the growth plate but not of the endosteal bone surface (25). Fluctuation in serum phosphorus concentration has been demonstrated with intermittent oral phosphorus loading, which may lead to reduced efficacy (26). The need for frequent oral administration with conventional therapy makes it less acceptable, leading to cessation of treatment in a substantial number of patients. Variations in conventional therapy protocols worldwide could be one of the confounding factors in assessing the efficacy of this treatment approach. This has been well illustrated in the study by Jin and cols., in which phosphorus with high-dose calcitriol (40 ng/kg/day) resulted in better improvement of rickets compared with low-dose calcitriol (20 ng/kg/day) (22). In addition, the height z-score is often on the lower side in patients with XLH receiving conventional therapy (10). Regarding the safety profile of phosphorus plus active vitamin D, it is very clear that XLH *per se* is not associated with nephrocalcinosis or renal calculi, unlike causes of hypophosphatemia independent from FGF23. Oral medical therapy has been demonstrated to increase the risk of nephrocalcinosis, and the risk is significantly higher in patients on high phosphorus dose (11). This is likely due to the delivery of a higher load of phosphorus to the kidneys in patients on conventional therapy. Additionally, phosphorus supplementation is associated with a risk of secondary and tertiary hyperparathyroidism (27). The current review also revealed the occurrence of secondary hyperparathyroidism in the conventional-therapy arm.

The analysis of studies involving adult cases revealed a beneficial effect of burosumab in normalizing phosphorus levels by stimulation of renal phosphorus absorption. Burosumab is more acceptable in cases with XLH, as it is administered subcutaneously every 4 weeks in adults. A study by Insogna and cols. showed complete healing of almost half of the fractures identified at baseline (14). In another study, these authors showed that burosumab significantly improved histomorphometric indices of osteomalacia (28). The improvements in osteomalacia were accompanied by an increase in serum phosphorus levels and biochemical markers of bone remodeling. Burosumab was well tolerated in adults and had no severe side effects.

The present systematic review has some limitations. First, it included only nine studies, with five of those in the pediatric population. Second, only one study included a head-to-head comparison of both treatments in pediatric patients. Third, due to inadequate data, absence of a placebo control group, and only a single study with two arms for comparison, a meta-analysis could not be performed. Fourth, the forest plot was created using pretreatment and post-treatment data; hence, a head-to-head comparison of burosumab versus conventional therapy could not be done. Fifth, the studies did not include infants, thereby limiting generalization. Sixth, no comparative trials are available in adults only, and the population in studies including adults may be representative of a subpopulation with more severe disease. A meta-regression was the foremost option but could not be conducted due to inadequate number of studies.

Despite the limitations, it is clear from the results of the present review that burosumab has several advantages over conventional therapy. The response was clearly greater with burosumab than with oral phosphorus and active vitamin D, although a direct head-to-head comparison could not be made. Burosumab targets the central pathophysiology of the disease, making it likely the most preferable agent with its superior efficacy, excellent safety profile, and sound therapeutic rationale. One major drawback is the limited accessibility to burosumab and its high cost, which restricts its widespread use in developing countries. Though sustained efficacy and safety for more than 3 years have been established in the study by Linglart and cols., more long-term prospective studies are required to confirm the safety profile over a longer period (29). We also suggest more studies in children younger than 1 year to prove the efficacy and safety of burosumab in this specific group.

In conclusion, the results of the present systematic review suggest a potential role for burosumab in improving rickets, deformity, and growth among children with XLH in different parts of the world. Furthermore, burosumab is effective in improving bone-related symptoms and facilitating fracture healing in adults with XLH. Given its superior efficacy and safety profile, burosumab could potentially replace conventional therapy as front-line therapy for children with XLH. Due to limited data, we were unable to draw a definitive conclusion concerning the efficacy and safety of burosumab in XLH. Further studies with robust design and inclusion of two arms (burosumab and conventional therapy) are warranted to produce conclusive evidence in both children and adults.

## Data Availability

the data that support the findings of this study are available from the corresponding author upon reasonable request.

## SUPPLEMENTARY

**Supplementary Table 1:** PRISMA 2020 checklist

Section and Topic	Item #	Checklist item	Location where item is reported
**TITLE**			
Title	1	Identify the report as a systematic review.	Page 1, Line 2
**ABSTRACT**			
Abstract	2	See the PRISMA 2020 for Abstracts checklist.	Page 1
**INTRODUCTION**			
Rationale	3	Describe the rationale for the review in the context of existing knowledge.	Page 2
Objectives	4	Provide an explicit statement of the objective(s) or question(s) the review addresses.	Page 2
**METHODS**			
Eligibility criteria	5	Specify the inclusion and exclusion criteria for the review and how studies were grouped for the syntheses.	Page 3
Information sources	6	Specify all databases, registers, websites, organisations, reference lists and other sources searched or consulted to identify studies. Specify the date when each source was last searched or consulted.	Page 3
Search strategy	7	Present the full search strategies for all databases, registers and websites, including any filters and limits used.	Page 3
Selection process	8	Specify the methods used to decide whether a study met the inclusion criteria of the review, including how many reviewers screened each record and each report retrieved, whether they worked independently, and if applicable, details of automation tools used in the process.	Page 3
Data collection process	9	Specify the methods used to collect data from reports, including how many reviewers collected data from each report, whether they worked independently, any processes for obtaining or confirming data from study investigators, and if applicable, details of automation tools used in the process.	Page 3
Data items	10a	List and define all outcomes for which data were sought. Specify whether all results that were compatible with each outcome domain in each study were sought (e.g. for all measures, time points, analyses), and if not, the methods used to decide which results to collect.	Page 4
	10b	List and define all other variables for which data were sought (e.g., participant and intervention characteristics, funding sources). Describe any assumptions made about any missing or unclear information.	Page 4
Study risk of bias assessment	11	Specify the methods used to assess risk of bias in the included studies, including details of the tool(s) used, how many reviewers assessed each study and whether they worked independently, and if applicable, details of automation tools used in the process.	Page 3
Effect measures	12	Specify for each outcome the effect measure(s) (e.g. risk ratio, mean difference) used in the synthesis or presentation of results.	Page 4
Synthesis methods	13a	Describe the processes used to decide which studies were eligible for each synthesis (e.g. tabulating the study intervention characteristics and comparing against the planned groups for each synthesis (item #5)).	Table 1
	13b	Describe any methods required to prepare the data for presentation or synthesis, such as handling of missing summary statistics, or data conversions.	Nothing specific
	13c	Describe any methods used to tabulate or visually display results of individual studies and syntheses.	"Escalc" function in Metafor
	13d	Describe any methods used to synthesize results and provide a rationale for the choice(s). If meta-analysis was performed, describe the model(s), method(s) to identify the presence and extent of statistical heterogeneity, and software package(s) used.	Random-effects model. We used standardized mean difference, as it is more generalizable
	13e	Describe any methods used to explore possible causes of heterogeneity among study results (e.g. subgroup analysis, meta-regression).	---
	13f	Describe any sensitivity analyses conducted to assess robustness of the synthesized results.	---
Reporting bias assessment	14	Describe any methods used to assess risk of bias due to missing results in a synthesis (arising from reporting biases).	---
Certainty assessment	15	Describe any methods used to assess certainty (or confidence) in the body of evidence for an outcome.	---
**RESULTS**			
Study selection	16a	Describe the results of the search and selection process, from the number of records identified in the search to the number of studies included in the review, ideally using a flow diagram.	Page 4
	16b	Cite studies that might appear to meet the inclusion criteria, but which were excluded, and explain why they were excluded.	Page 4
Study characteristics	17	Cite each included study and present its characteristics.	Table 1
Risk of bias in studies	18	Present assessments of risk of bias for each included study.	Supplementary Table 2
Results of individual studies	19	For all outcomes, present, for each study: (a) summary statistics for each group (where appropriate) and (b) an effect estimates and its precision (e.g. confidence/credible interval), ideally using structured tables or plots.	Table 2 and Figures 2 and 3
Results of syntheses	20a	For each synthesis, briefly summarise the characteristics and risk of bias among contributing studies.	Supplementary Table 2
	20b	Present results of all statistical syntheses conducted. If meta-analysis was done, present for each the summary estimate and its precision (e.g. confidence/credible interval) and measures of statistical heterogeneity. If comparing groups, describe the direction of the effect.	Table 2 and Pages 5 and 6
	20c	Present results of all investigations of possible causes of heterogeneity among study results.	-----
	20d	Present results of all sensitivity analyses conducted to assess the robustness of the synthesized results.	-----
Reporting biases	21	Present assessments of risk of bias due to missing results (arising from reporting biases) for each synthesis assessed.	-----
Certainty of evidence	22	Present assessments of certainty (or confidence) in the body of evidence for each outcome assessed.	-----
**DISCUSSION**			
Discussion	23a	Provide a general interpretation of the results in the context of other evidence.	Page 6
	23b	Discuss any limitations of the evidence included in the review.	Page 7
	23c	Discuss any limitations of the review processes used.	Page 7
	23d	Discuss implications of the results for practice, policy, and future research.	Page 8
OTHER INFORMATION			
Registration and protocol	24a	Provide registration information for the review, including register name and registration number, or state that the review was not registered.	Pages 2 and 3
	24b	Indicate where the review protocol can be accessed, or state that a protocol was not prepared.	Page 3 (PROSPERO registration)
	24c	Describe and explain any amendments to information provided at registration or in the protocol.	-----
Support	25	Describe sources of financial or non-financial support for the review, and the role of the funders or sponsors in the review.	None
Competing interests	26	Declare any competing interests of review authors.	None
Availability of data, code and other materials	27	Report which of the following are publicly available and where they can be found: template data collection forms; data extracted from included studies; data used for all analyses; analytic code; any other materials used in the review.	Data will be made available upon request to the corresponding author

Page MJ, McKenzie JE, Bossuyt PM, Boutron I, Hoffmann TC, Mulrow CD, et al. The PRISMA 2020 statement: an updated guideline for reporting systematic reviews. BMJ 2021;372:n71. doi: 10.1136/bmj.n71. For more information: http://www.prisma-statement.org/

**Supplementary Table 2:** Risk of bias assessment of the studies (n = 9) included in the systematic review

	Randomization process	Deviations from intended interventions	Missing outcome data	Measurement of the outcome	Selection of the reported result	Overall bias
Assignment to intervention (the "intention-to-treat" effect)						
Low risk of bias	50	100	100	87.5	100	50
Some concern	37.5	0	0	12.5	0	37.5
High risk of bias	0	0	0	0	0	0

## References

[1] Carpenter TO, Imel EA, Holm IA, Jan de Beur SM, Insogna KL (2011). A clinician's guide to X-linked hypophosphatemia. J Bone Miner Res.

[2] Carpenter TO, Shaw NJ, Portale AA, Ward LM, Abrams SA, Pettifor JM (2017). Rickets. Nat Rev Dis Primer.

[3] Gohil A, Imel EA (2019). FGF23 and Associated Disorders of Phosphate Wasting. Pediatr Endocrinol Rev.

[4] Beck-Nielsen SS, Mughal Z, Haffner D, Nilsson O, Levtchenko E, Ariceta G (2019). FGF23 and its role in X-linked hypophosphatemia-related morbidity. Orphanet J Rare Dis.

[5] Imel EA (2021). Congenital Conditions of Hypophosphatemia in Children. Calcif Tissue Int.

[6] Lee JY, Imel EA (2013). The changing face of hypophosphatemic disorders in the FGF-23 era. Pediatr Endocrinol Rev.

[7] Reid IR, Hardy DC, Murphy WA, Teitelbaum SL, Bergfeld MA, Whyte MP (1989). X-linked hypophosphatemia: a clinical, biochemical, and histopathologic assessment of morbidity in adults. Medicine (Baltimore).

[8] Linglart A, Biosse-Duplan M, Briot K, Chaussain C, Esterle L, Guillaume-Czitrom S (2014). Therapeutic management of hypophosphatemic rickets from infancy to adulthood. Endocr Connect.

[9] Mäkitie O, Doria A, Kooh SW, Cole WG, Daneman A, Sochett E (2003). Early treatment improves growth and biochemical and radiographic outcome in X-linked hypophosphatemic rickets. J Clin Endocrinol Metab.

[10] Zivičnjak M, Schnabel D, Billing H, Staude H, Filler G, Querfeld U (2011). Age-related stature and linear body segments in children with X-linked hypophosphatemic rickets. Pediatr Nephrol.

[11] Taylor A, Sherman NH, Norman ME (1995). Nephrocalcinosis in X-linked hypophosphatemia: effect of treatment versus disease. Pediatr Nephrol.

[12] DeLacey S, Liu Z, Broyles A, El-Azab SA, Guandique CF, James BC (2019). Hyperparathyroidism and parathyroidectomy in X-linked hypophosphatemia patients. Bone.

[13] Imel EA, DiMeglio LA, Hui SL, Carpenter TO, Econs MJ (2010). Treatment of X-linked hypophosphatemia with calcitriol and phosphate increases circulating fibroblast growth factor 23 concentrations. J Clin Endocrinol Metab.

[14] Insogna KL, Briot K, Imel EA, Kamenický P, Ruppe MD, Portale AA (2018). A Randomized, Double-Blind, Placebo-Controlled, Phase 3 Trial Evaluating the Efficacy of Burosumab, an Anti-FGF23 Antibody, in Adults with X-Linked Hypophosphatemia: Week 24 Primary Analysis. J Bone Miner Res.

[15] Carpenter TO, Whyte MP, Imel EA, Boot AM, Högler W, Linglart A (2018). Burosumab Therapy in Children with X-Linked Hypophosphatemia. N Engl J Med.

[16] Imel EA, Glorieux FH, Whyte MP, Munns CF, Ward LM, Nilsson O (2019). Burosumab versus conventional therapy in children with X-linked hypophosphataemia: a randomised, active-controlled, open-label, phase 3 trial. Lancet.

[17] Whyte MP, Carpenter TO, Gottesman GS, Mao M, Skrinar A, San Martin J (2019). Efficacy and safety of burosumab in children aged 1-4 years with X-linked hypophosphataemia: a multicentre, open-label, phase 2 trial. Lancet Diabetes Endocrinol.

[18] Carpenter TO, Imel EA, Ruppe MD, Weber TJ, Klausner MA, Wooddell MM (2014). Randomized trial of the anti-FGF23 antibody KRN23 in X-linked hypophosphatemia. J Clin Invest.

[19] Thacher TD, Fischer PR, Pettifor JM, Lawson JO, Manaster BJ, Reading JC (2000). Radiographic scoring method for the assessment of the severity of nutritional rickets. J Trop Pediatr.

[20] Lim R, Shailam R, Hulett R, Skrinar A, Nixon A, Williams A (2021). Validation of the Radiographic Global Impression of Change (RGI-C) score to assess healing of rickets in pediatric X-linked hypophosphatemia (XLH). Bone.

[21] Takeshima N, Sozu T, Tajika A, Ogawa Y, Hayasaka Y, Furukawa TA (2014). Which is more generalizable, powerful and interpretable in meta-analyses, mean difference or standardized mean difference?. BMC Med Res Methodol.

[22] Jin C, Zhang C, Ni X, Zhao Z, Xu L, Wu B (2022). The efficacy and safety of different doses of calcitriol combined with neutral phosphate in X-linked hypophosphatemia: a prospective study. Osteoporos Int J.

[23] Imel EA, Zhang X, Ruppe MD, Weber TJ, Klausner MA, Ito T (2015). Prolonged Correction of Serum Phosphorus in Adults With X-Linked Hypophosphatemia Using Monthly Doses of KRN23. J Clin Endocrinol Metab.

[24] Cheong HI, Yoo HW, Adachi M, Tanaka H, Fujiwara I, Hasegawa Y (2018). First-in-Asian Phase I Study of the Anti-Fibroblast Growth Factor 23 Monoclonal Antibody, Burosumab: Safety and Pharmacodynamics in Adults with X-linked Hypophosphatemia. JBMR Plus.

[25] Glorieux FH, Marie PJ, Pettifor JM, Delvin EE (1980). Bone response to phosphate salts, ergocalciferol, and calcitriol in hypophosphatemic vitamin D-resistant rickets. N Engl J Med.

[26] Glorieux FH, Scriver CR, Reade TM, Goldman H, Roseborough A (1972). Use of phosphate and vitamin D to prevent dwarfism and rickets in X-linked hypophosphatemia. N Engl J Med.

[27] Rivkees SA, el-Hajj-Fuleihan G, Brown EM, Crawford JD (1992). Tertiary hyperparathyroidism during high phosphate therapy of familial hypophosphatemic rickets. J Clin Endocrinol Metab.

[28] Insogna KL, Rauch F, Kamenický P, Ito N, Kubota T, Nakamura A (2019). Burosumab Improved Histomorphometric Measures of Osteomalacia in Adults with X-Linked Hypophosphatemia: A Phase 3, Single-Arm, International Trial. J Bone Miner Res.

[29] Linglart A, Imel EA, Whyte MP, Portale AA, Högler W, Boot AM (2022). Sustained Efficacy and Safety of Burosumab, a Monoclonal Antibody to FGF23, in Children With X-Linked Hypophosphatemia. J Clin Endocrinol Metab.

